# Reinventing Biostatistics Education for Basic Scientists

**DOI:** 10.1371/journal.pbio.1002430

**Published:** 2016-04-08

**Authors:** Tracey L. Weissgerber, Vesna D. Garovic, Jelena S. Milin-Lazovic, Stacey J. Winham, Zoran Obradovic, Jerome P. Trzeciakowski, Natasa M. Milic

**Affiliations:** 1 Division of Nephrology and Hypertension, Mayo Clinic, Rochester, Minnesota, United States of America; 2 Department of Medical Statistics and Informatics, Medical Faculty, University of Belgrade, Belgrade, Serbia; 3 Division of Biomedical Statistic and Informatics, Mayo Clinic, Rochester, Minnesota, United States of America; 4 Center for Data Analytics and Biomedical Informatics, Temple University, Philadelphia, Pennsylvania, United States of America; 5 Department of Medical Physiology, Texas A&M Health Science Center, Texas A&M University, College Station, Texas, United States of America

## Abstract

Numerous studies demonstrating that statistical errors are common in basic science publications have led to calls to improve statistical training for basic scientists. In this article, we sought to evaluate statistical requirements for PhD training and to identify opportunities for improving biostatistics education in the basic sciences. We provide recommendations for improving statistics training for basic biomedical scientists, including: 1. Encouraging departments to require statistics training, 2. Tailoring coursework to the students’ fields of research, and 3. Developing tools and strategies to promote education and dissemination of statistical knowledge. We also provide a list of statistical considerations that should be addressed in statistics education for basic scientists.

## Abstract 2

How is biostatistics education failing to meet the needs of basic scientists, and what can we do to fix this? This Perspective discusses strategies for making biostatistics an integral part of postgraduate and continuing education for basic scientists, ensuring that courses teach the specific skills needed to design, analyze, and critically evaluate basic science research.

## Introduction

Misuse of statistical methods is common in basic biomedical science research, even among papers published in high impact journals [1–3]. This includes using incorrect or suboptimal tests [1,2], summarizing data that were analyzed by nonparametric techniques as mean and standard deviation or standard error [4], reporting *p*-values that are inconsistent with the test statistic [5,6], p-hacking [7], and analyzing nonindependent data as though they are independent [3]. Additional problems arise from inadequate reporting of statistical methods. This may include failing to provide a power calculation [1], not reporting which statistical test was used, or not providing adequate detail about the test (i.e., paired versus unpaired *t* test) [1], not addressing whether the assumptions of the statistical tests were examined [1,4], or not specifying how replicates were treated in the analysis [3]. Finally, other researchers have focused on the need to reconsider current statistical practices. The reliance on null hypothesis testing and *p*-values has been heavily questioned, and researchers have proposed a variety of alternate approaches [8,9]. These problems stem from a limited understanding of statistics, suggesting that scientists need better training in this important skill set [10].

This article focuses on rethinking our approach to biostatistics education. Data from our previous systematic review of physiology studies [4] demonstrate that understanding statistical concepts and skills is essential for those who are reading or publishing scientific papers. We sought to determine whether this is reflected in the curriculae for PhD students by examining statistics education requirements among PhD programs in top NIH-funded physiology departments (*n* = 80). We then outline several approaches that may help to reinvent statistical education for basic biomedical scientists. Some of the problems that we discuss in this article are common to many fields, whereas other problems may require field-specific solutions. This article includes general recommendations based on the authors’ experiences in basic biomedical research. We hope that these comments will advance the ongoing discussion about improving the quality of data presentation and statistical analysis in basic science.

## Recommendation 1: Encourage Departments to Require Statistics Training

Data presentation and statistical analysis are increasingly important parts of scientific publications. This trend is likely to accelerate as more journals implement checklists to address common statistical problems and enlist statistical consultants to review papers [11,12]. The data presented in Box 1 show that the ability to understand statistical concepts and apply statistical skills is essential for research; however, biostatistics training is not always required to complete a PhD. We recommend that biostatistics be required for all doctoral students in disciplines where statistics are routinely used. Early career investigators who did not take a biostatistics course during their PhD training should obtain statistical training during their postdoctoral or early faculty years. This parallels recommendations from a recent Nature Medicine editorial [13], which emphasized that proper training in statistics and research methods is essential for reproducible research. The authors recommended that training in statistics and research methods be required for first year graduate students at PhD-granting institutions.

**Box 1. Statistical Skills Are Essential but Not Always Required for a PhD**

**Fig 1:**
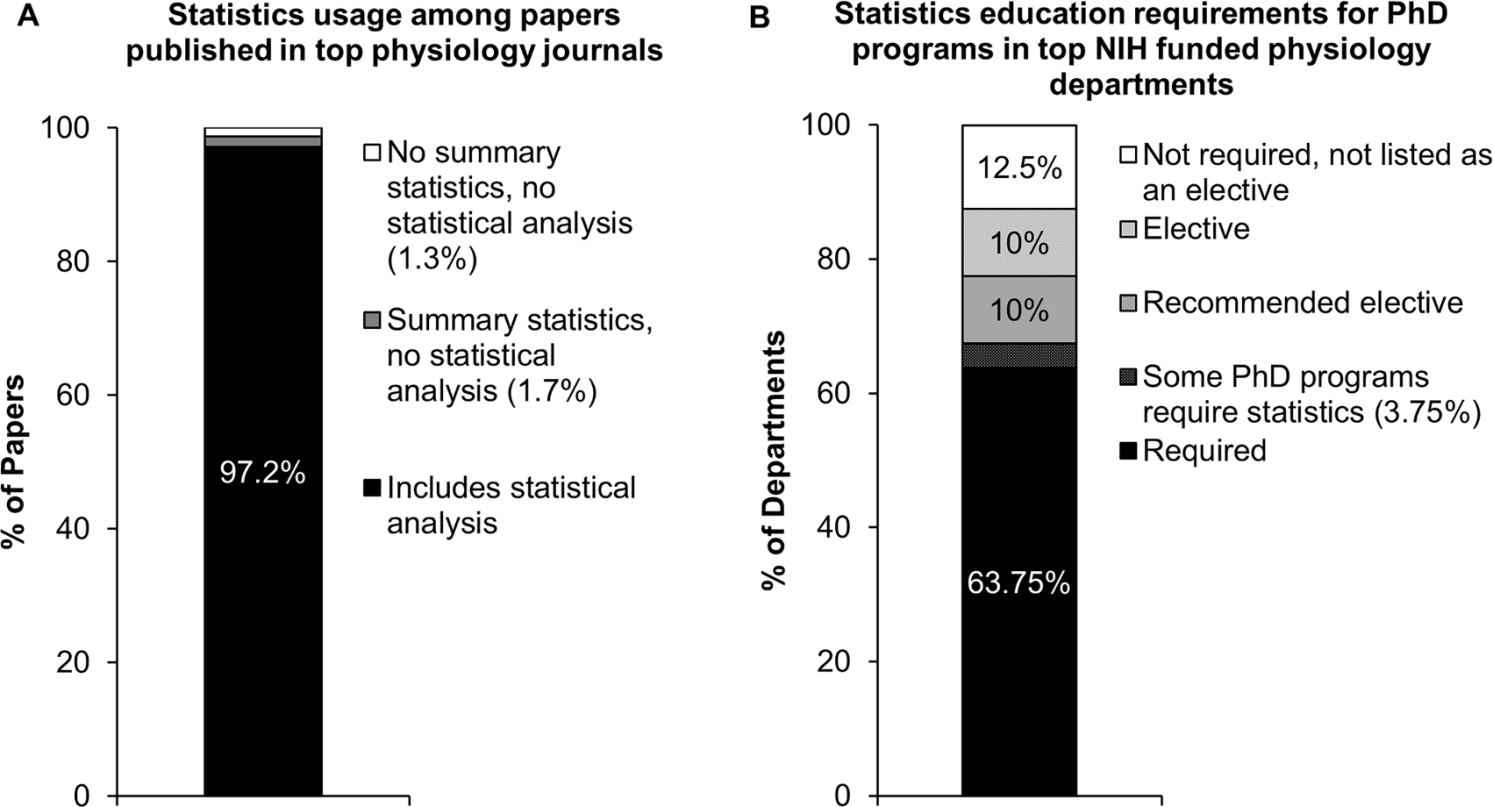
Statistics usage and education in physiology. **A:** A recent systematic review [4] demonstrated that 97.2% of papers published in the top 25% of physiology journals included statistical analyses. **B:** Statistics courses are not always required for PhD students in top NIH funded physiology departments. Detailed methodology for panels A and B are described in S1 Text.

- According to our recent systematic review [4], 97.2% of original research papers published in the top 25% of physiology journals (*n* = 683/703) included some form of statistical analysis (Fig 1A). Journals were selected based on 2012 impact factors.
- While this systematic review focused on physiology, frequent use of statistics likely extends to related disciplines. Physiology journals publish articles from researchers in many fields, including biochemistry, microbiology, cell biology, neuroscience, and many others.
- Among top NIH-funded physiology departments (*n* = 80), 67.5% required a statistics course for some (3.75%) or all (63.75%) PhD programs in which the department participated (Fig 1B). Biostatistics was recommended as an elective in 10% of departments and listed as an elective in 10% of departments. Biostatistics was not required or offered as an elective course for students in 12.5% of departments. This included one department that required a mathematical modeling course with a small biostatistics section. Departments included in this analysis were on the Blue Ridge Institute for Medical Research list of top NIH-funded physiology departments for 2014 (methodology in S1 Text).
- Students were rarely required to have learned statistics prior to starting a PhD program. One department (1.3%) listed a statistics course as a prerequisite for admission to the PhD program. Five departments (6.2%) recommended a statistics course prior to admission. No program required students to complete a Masters degree before entering the PhD program.
- Some departments offer PhD programs that are focused on physiology (*n* = 33), whereas others participate in departmental or interdepartmental PhD programs that include the related disciplines of biophysics, neuroscience, pharmacology or biology (*n* = 47). Statistics requirements were not different in a sensitivity analysis in which we excluded programs that combined physiology with related disciplines (Physiology only: *n* = 33).

## Recommendation 2: Tailoring Coursework to the Student’s Field of Research

While many departments currently offer or require biostatistics training, courses may not necessarily be designed to meet the needs of basic science students. This section focuses on strategies for designing courses that will give students the conceptual understanding and skills needed to analyze data, critique the literature, and improve the quality of statistical reporting and analysis in their respective fields.

### Lost in Translation: Bridging the Communication Gap between Basic Scientists and Statisticians

We propose that the faculty of basic science departments improve the quality of statistics education by working with statistics instructors to ensure that courses prepare students to read and publish papers in their respective fields. Among departments that include statistics as a required or elective course, many “out-source” their statistics teaching to other departments that offer introductory statistics courses. At some institutions in our sample (see Box 1, methodology in S1 Text), outside departments offered courses that appeared to be designed for basic scientists. At other institutions, introductory statistics courses designed for epidemiologists or public health students were incorporated into the basic science curriculum. The latter courses are unlikely to provide appropriate statistical preparation for basic scientists given the obvious differences in study designs and sample sizes between these disciplines.

Statisticians have recently questioned whether general introductory courses based on a one-size-fits-all approach to statistics education meet the needs of students [14]. Statistics is an increasingly specialized field, in which the techniques that are used vary widely depending on the type of outcome variable (continuous, categorical, time to event, etc.), the sample size, and the study design. Survival analyses, tests of predictive accuracy, odds ratios, and relative risks are common in clinical science but are rarely used by basic biomedical scientists, who typically work with continuous data, counts, or proportions. While introductory statistics courses generally focus on techniques for analyzing these types of data, the techniques and strategies that are taught often assume a much larger sample size than we observed in our systematic review of physiology studies.

Statisticians who teach general statistics courses often work with very large datasets and may have limited knowledge of the sample sizes or study designs that are common in basic biomedical research. These instructors might design their courses quite differently if they understood the characteristics of the datasets with which their students were working. We propose that the faculty in basic biomedical science departments collaborate with statistics instructors to ensure that courses teach students the skills that they will need to understand, present, and analyze data in their respective fields. Courses should be designed around the sample sizes, study designs, and types of data that are frequently used in the students’ areas of study. Coursework should also address errors in statistical analysis and data presentation that are common among published papers for that field. The following sections provide information about particular areas where the needs of basic scientists may differ from the concepts and skills that are generally taught in introductory statistics courses.

### Statistics for Small Samples

Small sample sizes are common in many basic science disciplines. Basic scientists typically want to compare values obtained from participants, specimens, or samples in different groups (i.e., wild-type mice versu knock-out mice; participants randomized to an exercise intervention versus a control group, men versus women), or at different time-points or conditions (i.e., preintervention versus postintervention, etc.). In a systematic review of papers published in top physiology journals [4], the median for the smallest sample size of any group shown in a figure was 4 (25th percentile: 3, 75th percentile: 6). The median sample size for the largest group shown in a figure was 9 (25th percentile: 6, 75th percentile: 15). Low statistical power and small sample sizes have been highlighted as one factor that may contribute to irreproducibility in neuroscience [15]. A recent study suggested that *n* = 8/group was a common sample size for preclinical research [16]. Anecdotal reports suggest that many investigators consider *n* = 6/group to be sufficient for animal studies, although this assumption is not based on statistical principles [17].

There are several possible reasons why researchers use small sample sizes. Experiments with large sample sizes are not feasible in many cases because the detailed mechanistic studies performed by basic scientists are very time, labor, and cost-intensive. The International Guiding Principles for Biomedical Research Involving Animals require that “the minimum number of animals should be used to achieve the scientific or educational goals” [18]. Finally, the variability in an experiment performed on a particular cell line or strain, type, or breed of animal may be lower than would be expected in a human study. Cell lines or animal studies sometimes lack diversity in genetic, environmental, and behavioral factors that can affect study measurements and increase variability in human studies.

Training for researchers working in fields where small sample sizes are common should focus on experimental design and statistical analysis considerations for small datasets. Students should learn that it is always better to show the actual data points instead of nonrepresentative summary statistics. While some types of studies are heavily dependent upon statistical analysis, other types of studies may not require statistical tests [10,19]. Investigators need to know how to distinguish between these two scenarios and learn how to present and interpret data in cases where statistical tests are not required. Alternative techniques, such as effect size indices with 95% confidence intervals, may be particularly valuable for small datasets [8].

In cases where power calculations and statistical analysis are needed, students should learn that sample size is one determinant of statistical power, along with effect size and variability. Power calculations often focus on avoiding false negative findings [8]. Students should understand that underpowered studies produce unreliable *p*-values and appreciate the risk and consequences of obtaining a false positive finding [8]. Underpowered studies are one factor that contributes to irreproducibility. Students should be able to determine whether it is feasible to conduct an adequately powered study to answer their research question. This may include performing their own power calculations, consulting with a biostatistician, and determining whether changes in the study design or outcome measurements would improve power. Students should learn how to select a new research question if an adequately powered study is not feasible. The analysis section of the course should focus on techniques for presenting and analyzing small sample size data (Box 2). Students should also understand why techniques that are commonly used for large datasets may not be appropriate for small datasets.

**Box 2. Statistical Considerations for Basic Scientists**

This list highlights areas where the needs of basic scientists may differ from material that is typically taught in introductory statistics courses.

1. Considerations for small sample size data*
  - Describing data: When are datasets too small for summary statistics, and what alternate methods should be used?
  - Probability and distributions: When are tests for a normal distribution too underpowered to be useful?
  - Statistical analysis: Alternative strategies for small sample size studies.
2. Strategies for identifying outliers and spurious data; understanding the consequences of deleting outliers that are not spurious data
3. Clustered Data
  - Understand clustered designs
  - Simple analysis strategies and their limitations
  - Know when more sophisticated analyses are required and how to consult a statistician
4. Selecting statistical software that promotes reproducible research
5. Presenting data in scientific presentations and publications
  - When to use figures versus tables
  - Selecting the right figure for the type of outcome variable (categorical, continuous, etc.), sample size, and study design
  - Choosing figures that show the distribution of continuous data instead of bar graphs
6. Critical evaluation of the literature: Students should be able to identify common problems with the way that data are presented and analyzed in published papers in their field and discuss solutions with colleagues and reviewers.
7. Know when and how to consult a statistician
8. Additional considerations: Bootstrapping and permutation tests, Bayesian statistics

* Note to statisticians: In basic biomedical science, a small sample size refers to groups consisting of fewer than 10…not fewer than 100 or 1,000. Sample sizes of 3 to 6 independent observations per group are very common [4].

### Strategies for Handling Attrition and Outliers

Basic scientists should have training in best practices for reporting attrition, identifying outliers or spurious data points, and analyzing datasets with these features. A recent meta-analysis highlighted the problems with reporting of attrition in preclinical animal studies of cancer and stroke [16]. The authors could not determine whether animals were excluded or did not complete the experiment in 64.2% of stroke studies and 72.9% of cancer studies. Among studies with clear evidence of attrition (the sample sizes in the methods and results section did not match), most authors did not explain the reasons for attrition.

Basic scientists often work with small samples [4,15,16], making it difficult to determine the data distribution and identify outliers. Simulation studies indicate that biased exclusion of a few animals can dramatically inflate the estimated treatment effect [16]. The potential for effect size inflation was particularly strong for small sample size studies, which are common in preclinical research, or when the authors excluded outliers that worked against the hypothesized treatment effect. Scientists working in fields with slightly larger sample sizes may benefit from learning resampling methods, such as bootstrapping methods and permutation tests. Resampling methods can be particularly relevant in experimental studies, because they do not assume a particular type of sampling distribution but estimate the sampling distribution empirically from the observed data.

### The Declaration of Dependence: Reporting Clustered Data

Knowing whether the data are independent is a critical consideration when selecting statistical tests. Techniques such as *t* tests, Wilcoxon rank-sum tests, and ANOVA, rely on the assumption of independence. Data are independent when the investigators perform one measurement in each subject or specimen, and the subjects or specimens are not related to each other. In contrast, techniques such as paired *t* tests, Wilcoxon signed-rank tests, and repeated measures ANOVA, assume that the data are not independent. In this design, the investigators repeat measurements on the same subject or specimen under more than one condition or time point. Repeated measures data are not independent and therefore require different analysis techniques.

Introductory statistics courses for basic scientists teach students to analyze independent data and often teach simple techniques for analyzing repeated measures data. Few introductory statistics courses teach students about clustered designs. Only one measurement is performed on each subject or specimen in clustered studies [20]; however, the data form nonindependent clusters, because some of the subjects or specimens are related to each other. Experiments with clusters of related subjects or specimen are common in physiology and many other basic sciences. Problems with the analysis and presentation of clustered data have been reported in neuroscience [3], toxicology [21], wound healing studies [22], psychology [23], and ecology [24]. In vitro laboratory studies, for example, typically include three independent experiments with two or three replicates per experiment. Replicates within an experiment should be more similar to each other than to values obtained during an independent experiment, as replicates are run on the same day, with the same reagents, using identical or nearly identical processing times and conditions. Clustered designs are also used in animal studies. If an investigator studies 30 neurons obtained from three different animals, then all neurons obtained from the same animal form a cluster of nonindependent data. If an investigator examines 25 newborn mice from four different litters, then data obtained from mice in the same litter are clustered.

Clustered designs are rarely included in introductory statistics courses, as procedures for analyzing clustered data can be quite complex. Many basic biomedical scientists do not recognize that these designs include nonindependent data [3]. This leads to confusion in the published literature. Some authors do not specify how clustered data were analyzed, whereas others analyze the data as though all points were independent. We strongly recommend that statistics instructors discuss clustered designs in courses for students working in fields where clustered data are common. Students should be able to recognize clustered designs, discuss the statistical implications of working with clustered data, and know when to consult a statistician. Understanding the statistical complexities associated with clustered designs may encourage students to avoid these designs in situations where simpler independent study designs are feasible.

### To Code or Not to Code…That Is the Question

While statisticians prefer statistical programs that require coding, most introductory statistics courses for basic scientists teach students to use programs that have a user-friendly interface. These programs make statistics less intimidating by allowing students to quickly learn how to perform basic statistical tests. Basic biomedical scientists often feel that programs that require coding are too complex and that the extra features that these programs offer are unnecessary for the simple analyses that are common in basic science (i.e., *t* tests, ANOVA).

Statistics programs with a user-friendly interface will likely continue to be a centerpiece of statistics education for basic biomedical scientists. However, there are several important considerations that should be taken into account when selecting statistical software for introductory statistics courses.

1. The ability to reuse code enhances reproducibility and saves time: Analyses run in coding-based programs are more reproducible. Researchers can save the code for each analysis, which can easily be rerun and checked by others. Reproducibility is more difficult to check in programs that have a user interface, as the results depend on the series of options selected by the user. A small change in any of these options can alter the results of the analysis. Investigators who consistently perform certain types of analysis can reuse code that they have previously written for similar analyses. This saves time, which can offset the additional time required when learning to use a coding-based program.
2. Cost and accessibility: Most universities and research centers purchase an institutional license for a particular statistics program with a user interface, then build their courses around that program. Trainees and junior investigators may move several times during the course of their careers. Statistics programs vary among institutions; therefore, young investigators often need to choose between purchasing an expensive individual license or learning to use a new statistics program each time that they move. There are several code-based software packages, in contrast, that are available to everyone free of charge. Researchers trained in these programs can continue to use their existing skills and develop new ones regardless of where they move.
3. Ability to run more complex analyses: Multidisciplinary research training is becoming increasingly common, and young investigators may transition among different fields or specialties early in their careers. Coding-based programs allow for more complex analyses. Researchers who are trained to use these programs will have a better foundation for expanding their skills should they move to a different discipline or work with datasets that require more advanced statistics.
4. Promoting knowledge retention: Programs that have a user interface often make decisions about what test to use based on the characteristics of the data. Investigators who use these programs may have less knowledge of the specific tests that are being performed or how they are implemented in the statistical software package. Coding-based programs require the user to know what test is needed. This may promote better retention of statistical knowledge.

Many basic biomedical scientists remain strongly opposed to software packages that require coding; however, several PhD programs in our sample offered required or elective statistics courses that were teaching students to use R, a code-based free-of-charge statistical program. Scientists who are trained to use R have access to an extensive library of packages designed for more complex analyses (available from http://cran.us.r-project.org/). These packages are available gratis and continue to evolve to meet the growing needs of R users. Other software programs may not offer these types of specialized analysis packages or may require the user to purchase modules for advanced statistical techniques separately.

### Data Presentation: Pretty Is Not Necessarily Perfect

Statistical education should include training in all stages of research, including sessions on designing tables and figures for publications. These are less common and, when offered, sometimes focus on making figures that are visually appealing. A visually appealing figure is of little value if it is not suitable for the type of data being presented.

Our systematic review of physiology studies discusses data presentation in more detail [4]. Students should learn how to decide which data to present in tables versus figures and how to select the appropriate type of figure for their data, based on the type of variable (continuous versus categorical), the study design, and the sample size. Information on how to design visually effective tables and figures can be added once students have learned these fundamentals. Students who do not receive training in data presentation are likely to consult colleagues or refer to published papers when they need to create figures, which may lead them to adopt standard practices for the field. This is a problem, as our recently published systematic review demonstrates that we urgently need to change the way that we present data in small sample size studies [4]. Data presentation training is essential to improve the quality of data presentation in the scientific literature.

### Critical Evaluation of the Literature

Statistics courses should include a “synthesis” module, where students discuss and critique data presentation and analysis practices for papers published in their respective fields. This module should include experiences with critical evaluation of individual papers, as well as a review of metaresearch articles that examine strengths and weaknesses of data presentation and analysis in related fields of research. Students should understand standard practices for their fields, be able to explain the problems with these practices, discuss the advantages and disadvantages of potential solutions, and select the solution that best fits a particular dataset. The benefits of statistics training may be lost if students are not trained to address common problems with standard data presentation and analysis practices in their field of research.

### Knowing When and How to Consult a Statistician

While this paper focuses on biostatistics training for basic scientists, we strongly support initiatives to build collaborations between basic scientists and biostatisticians. An effective statistics course not only teaches students what they have learned; it should also teach students what they have not learned. Basic scientists should be able to identify common situations in which statistical tests are not appropriate and know when the study team lacks the expertise to perform more complex analyses that may be required. They should be prepared to consult with a statistician to determine whether a different study design can be used, and plan for any statistical resources that will be needed. Basic scientists should be aware of institutional resources that provide statistical support, understand the complementary roles of basic scientists and statisticians, and learn how to develop and maintain an effective collaboration with a statistician during all stages of the research process.

## Recommendation 3: Develop Tools and Strategies to Promote Education and Dissemination of Statistical Knowledge

### Establishing the Importance of Statistics in the Student’s Field of Study

Students and professors sometimes feel that statistics courses are less valuable than core courses in the student’s chosen field. Although statistics is an essential skill, medical students reported neutral perceptions about the value of biostatistics and their interest in statistics [25]. The perception that statistics is intimidating, or that statistics courses are not relevant to the student’s field of study can interfere with student efforts to learn the statistical concepts and skills that they will need to conduct sound research. Several strategies discussed in this article may help to mitigate this problem. Courses centered around field-specific study designs, datasets, and exercises implicitly show students how statistical knowledge is critical to their research careers. General courses in which topics and materials do not align with research conducted in the students’ field of study may inadvertently suggest that statistics are less relevant and less important. Critiquing the literature will help students to appreciate the importance of statistics in conducting reproducible research in their chosen fields. Finally, we suggest that mentors meet with mentees to discuss the role of biostatistics in the mentee’s field of study and to emphasize the ways in which understanding biostatistics skills and concepts is crucial to success.

### Customizing Statistics Education with Limited Resources

Tailoring courses to the needs of students in different fields of study is labor intensive and may not be feasible at institutions with a limited number of statistics instructors. Integrating customized online modules into general statistics courses may be one potential solution to this problem. Neuroscientists, for example, might complete a module on clustered data, whereas geneticists might complete a module on quality control. This approach would allow trainees to learn field-specific concepts and skills, without requiring a separate statistics course for each department. A comprehensive meta-analysis recently reported that well-organized online classrooms were as effective and of comparable quality to traditional classrooms [26]. Furthermore, there was a modest improvement in learning in courses that combined online learning with face-to-face instruction, when compared to face-to-face instruction alone [26]. One of the authors (NMM) has successfully used this combined approach to teach medical statistics [27].

A second option would be for professional societies to facilitate customized statistics education by defining core competencies and creating educational materials. This might include field-specific sample datasets and exercises, as well as online modules for topics that are not routinely included in general statistics courses. These materials could be integrated into existing courses or viewed independently by established investigators seeking additional training on particular topics. The National Institutes of Health Rigor and Reproducibility Training Modules provide an example of this “open access” approach to providing educational materials (http://1.usa.gov/1OmBIWZ). Field-specific materials may also be a useful strategy for increasing trainees’ interest in statistics.

### Do One, Teach One: Improving Statistical Knowledge in the Scientific Community (Dissemination)

We propose that statistics courses should prepare students to disseminate their knowledge to others in their field. Formal statistics education typically targets trainees and junior investigators. Senior investigators play a much more prominent role in shaping the literature; however, most do not have time to take courses. Offering online lectures for continuing professional development would allow investigators at all levels to augment their knowledge of current topics in statistics and data presentation. A “grass-roots” approach to statistics education is also needed, as many trainees and junior scientists will need to convince peers, colleagues, and reviewers if they want to improve the quality of data presentation and statistical analysis in published papers. In addition to completing the “synthesis” module that was described previously, students should also know key references that describe problems with the standard practices in their field and outline solutions. References that focus on the practical implications of statistical techniques and are accessible to readers with little or no statistics background may be most valuable in encouraging basic scientists to re-evaluate their approach to data presentation, statistical analysis, and statistics education.

## Conclusions

Although understanding statistical concepts and skills is essential for basic science research, biostatistics training is not always required to complete a PhD. Our recommendations for improving statistics training for basic biomedical scientists include: 1. Encouraging departments to require statistics training, 2.Tailoring coursework to the student’s field of research, and 3. Developing tools and strategies to promote education and dissemination of statistical knowledge. Faculty members of basic science departments should work with statistics instructors to design coursework that focuses on the study designs, types of outcomes, and sample sizes that are common in the students’ field. Finally, students should learn to critically evaluate data presentation and statistical analysis in the published literature.

## Supporting Information

S1 Fig Flow chart for systematic review of physiology studies. (TIF)

S2 Fig Flow chart for study of statistical education practices in PhD programs. (TIF)

S1 Text Supplemental methods and results for the data presented in Box 1 and Fig 1. (DOCX)
