# Supplementary figures and images for: Reinventing Biostatistics Education for Basic Scientists
Source: PLoS Biol. 2016 Apr 8;14(4):e1002430. doi: 10.1371/journal.pbio.1002430 (PMC4825954; doi:10.1371/journal.pbio.1002430)

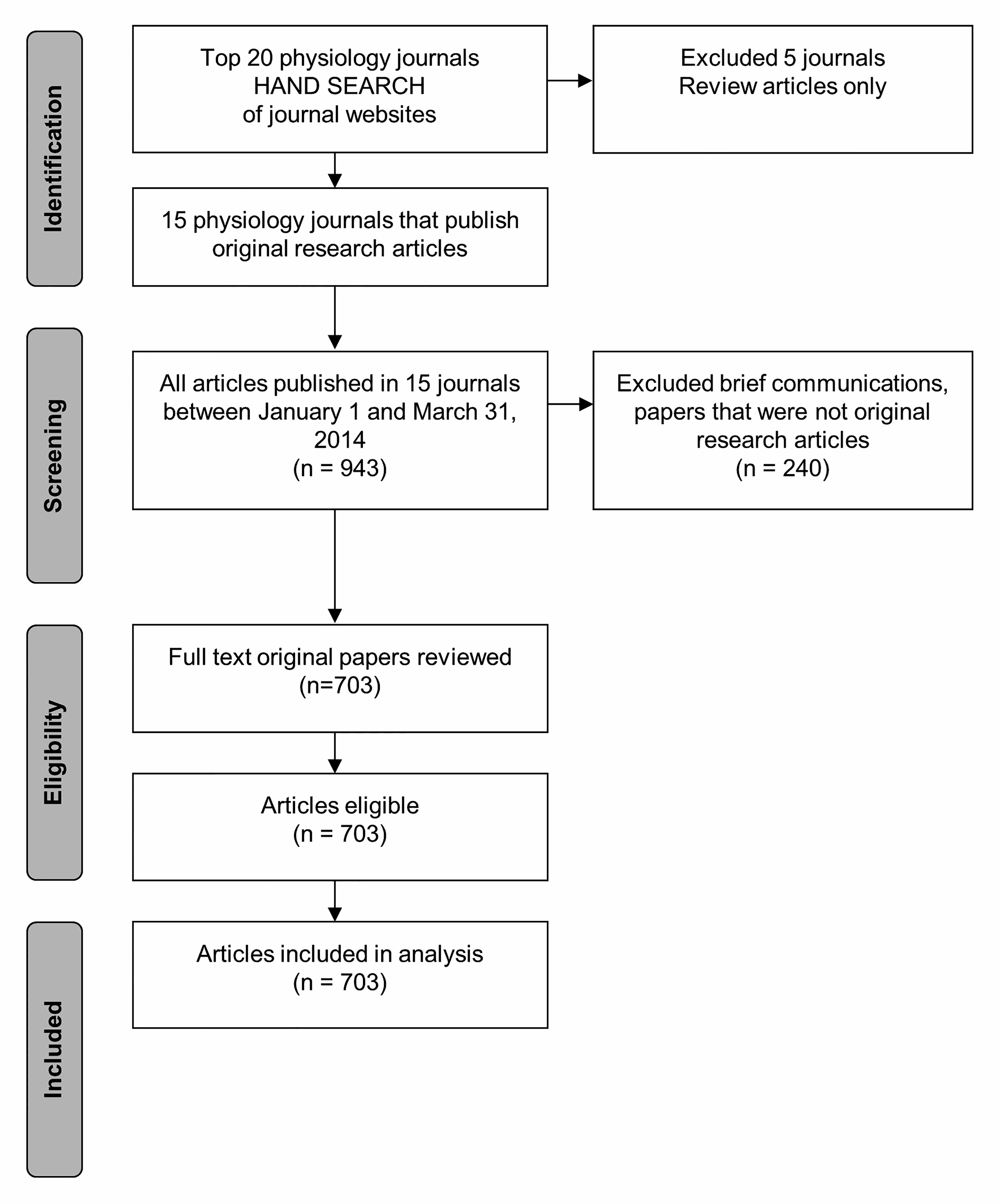

Supplement: S1 Fig — (TIF) [file pbio.1002430.s001.tif]

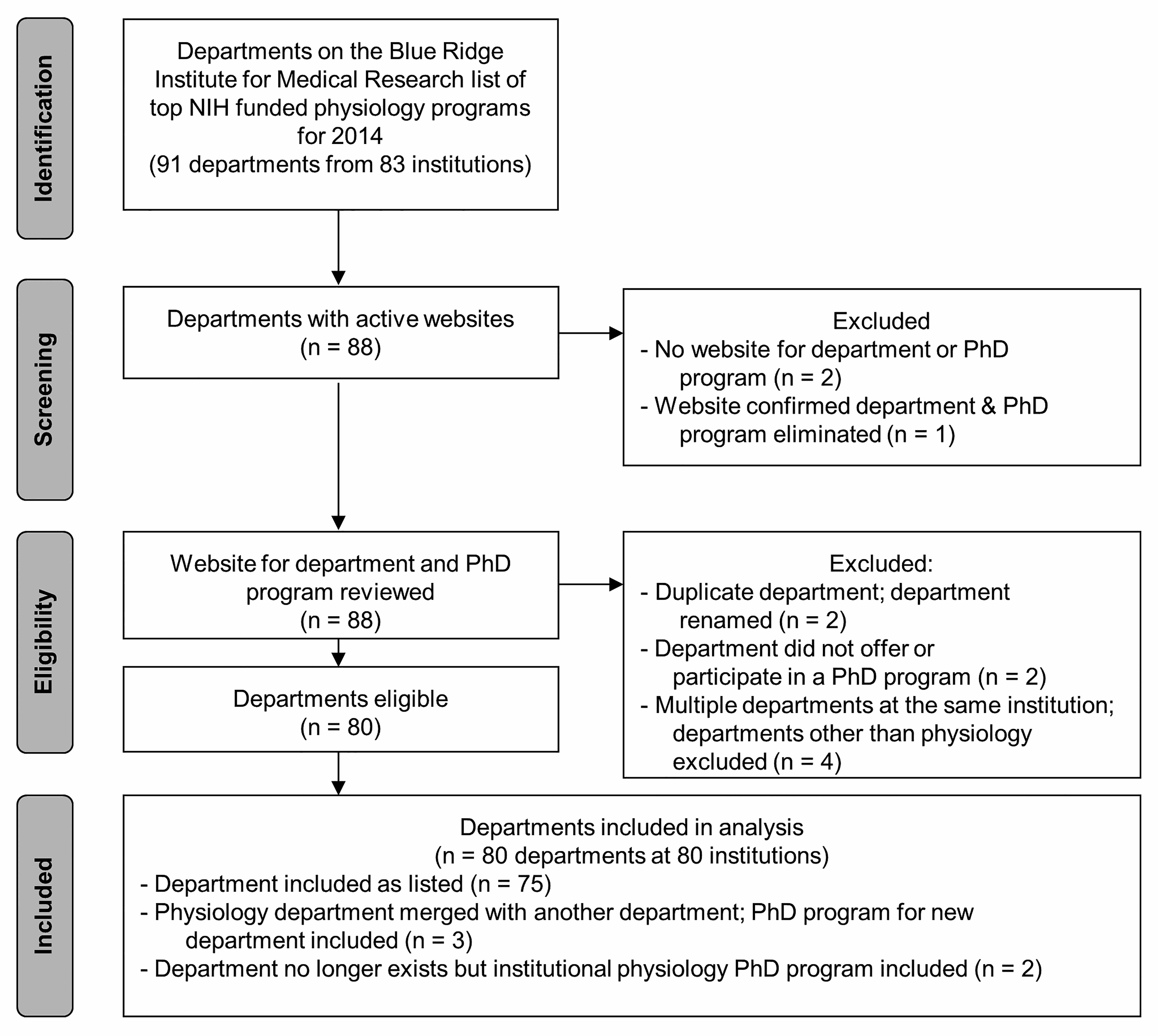

Supplement: S2 Fig — (TIF) [file pbio.1002430.s002.tif]
