# Supplementary material for: Reinventing Biostatistics Education for Basic Scientists
Source: PLoS Biol. 2016 Apr 8;14(4):e1002430. doi: 10.1371/journal.pbio.1002430 (PMC4825954; doi:10.1371/journal.pbio.1002430)
Supplement: S1 Text — (DOCX) [file pbio.1002430.s003.docx]

**Methods**

**Systematic Review**

Our previous paper describes detailed methods for the systematic review [1]. Briefly, we examined all original research articles published in the top 25% of physiology journals between January 1 and March 31, 2014 (n = 703). Journal rankings were determined based on 2012 impact factors. Two independent reviewers assessed each article. Reviewers classified each article into one of three mutually exclusive categories: 1. The article included any form of statistical analysis, 2. The article presented summary statistics but did not include any statistical analyses, or 3. The article did not include summary statistics or statistical analyses. For papers that did not include summary statistics or statistical analyses, reviewers also determined whether the paper involved mathematical modeling. Disagreements were resolved by consensus.

**Review of Statistics Education Requirements in Top NIH-funded Physiology Departments**

**Ethics Statement:** The Mayo Clinic Institutional Review Board classified this research as exempt (15-003651).

**Methods:** We examined websites for departments on the Blue Ridge Institute for Medical Research list of top NIH funded physiology departments for 2014 (n = 80, http://www.brimr.org/NIH_Awards/2014/NIH_Awards_2014.htm). Two reviewers independently reviewed websites for each department to determine:

1. Whether the department offered it’s own PhD program, or participated in an interdepartmental PhD program. Some departments participated in multiple PhD programs. If the department website noted that one or more programs were run by the department or that most students in the department enrolled in particular program(s), we only abstracted information for those program(s). If the department website did not provide any guidance about what program their students usually enrolled in, we abstracted information for all PhD programs.

2. Whether the PhD program required a Masters degree or statistics course for admission

3. Whether a biostatistics course was required for completion of the PhD program. If a statistics course was not required, we determined whether a statistics course was recommended, listed as an optional elective or not required and not listed as an elective. If a statistics course was required, recommended or listed as an optional elective, we determined the course name and the name of the department that offered the course.

4. If a biostatistics course was not required, we determined whether a statistical course in bioinformatics, genetic analysis or mathematical modelling was required for completion of the PhD (data not shown).

5. Websites from which information was obtained and the date on which websites were accessed.

To verify the information obtained from the department website, we emailed the each department head and graduate coordinator on two occasions. If there was no response, we called the department office to request contact information for someone who could verify the information before sending two follow-up emails.

The goal of this research was to improve our understanding of statistics education requirements for PhD programs in physiology departments; not to highlight practices in particular departments. As such, our IRB protocol specifies that data will only be presented in aggregate. The authors can provide a limited dataset without identifying institutional information upon request for readers who wish to confirm the results of our analyses.

**Results**

**Statistics Usage Among Papers Published in Top Physiology Journals:** Our systematic review included 703 original research articles published in the top 25% of physiology journals between January 1 and March 31, 2014. The flowchart in Figure S1 illustrates the inclusion of studies for the present analysis. 97.2% of papers (n = 683) included some form of statistical analysis (Figure S1). 1.7% (n = 11) included summary statistics but did not perform any statistical analyses. The remaining 1.3% (n = 9) did not include any summary statistics or statistical analyses. Among these 9 papers, 8 used mathematical modeling techniques.

**Statistics Education in Top NIH Funded Physiology Departments:** The Blue Ridge Institute for Medical Research list of top NIH funded physiology departments for 2014 included 91 departments from 83 institutions (Figure S2). One institution was excluded because the department website confirmed that the department and PhD program had been eliminated. Two departments were excluded because no website could be found, suggesting that these departments no longer existed. Websites for the remaining 88 departments were screened to determine whether each department offered or participated in a PhD program. Two departments had been renamed and both the old and new department names appeared on the list. The duplicate entry for the old department name was excluded. Two departments were excluded because they did not offer or participate in a PhD program. Four departments were excluded because the list included multiple departments from the same institution; in these cases we included only the physiology department. The four excluded departments included two biophysics departments and two research institutes for specific conditions or physiological systems.

Eighty departments from 80 institutions were eligible for the analysis. In 75 cases, we abstracted information for the department as named on the Blue Ridge Institute for Medical Research list. In three cases, the physiology department had merged with another department. We abstracted information for the PhD program offered by this new department (n = 3). Two departments had been disbanded; however, the institutions continued to offer a PhD in physiology. In these cases, we abstracted information for the institutional physiology PhD program (n = 2).

Among top NIH-funded physiology departments (n = 80), 67.5% required a statistics course for some (3.75%) or all (63.75%) PhD programs in which the department participated in (Figure 1, Panel B). Biostatistics was recommended as an elective in 10% of departments and listed as an elective in 10% of departments. Biostatistics was not required or offered as an elective course for students in 12.5% of departments. This included one department that required a mathematical modeling course with a small biostatistics section.

70 (87.5%) of departments responded to our requests to verify the information obtained from their websites. Compared to the information abstracted from the department or PhD program websites, 24 (34.3%) of departments reported a change in biostatistics requirements for their PhD program(s). This included six departments for which statistics requirements could not be determined from the department or PhD program websites. Changes for the remaining programs were due to recent updates to graduate program requirements, incomplete or inaccurate website information or the exclusion of PhD programs that students in the physiology department rarely or never enrolled in. In all cases where statistics requirements changed with verification, the department reported more stringent requirements compared to our initial classification. Statistics requirements were similar in a sensitivity analysis that excluded the 10 departments that did not respond to requests for verification (Statistics required: 62.9%, Statistics required for some programs: 4.3%, Recommended elective: 11.4%, Elective: 8.6%, Not required, not listed as an elective: 11.4%).

**Sensitivity Analysis of Physiology PhD programs:** While some departments offered PhD programs in physiology, others participated in interdepartmental PhD programs that included the related disciplines of biophysics, neuroscience, pharmacology, or biology. Statistical skills may differ between these different disciplines. We therefore identified a subgroup of PhD programs that focused on physiology by excluding all programs with the words “biophysics”, “neuroscience”, “pharmacology” and “biology” in the PhD name. PhD names for programs in this subgroup either had PhD programs entitled “physiology” or “physiology” in combination with one or more of the words “molecular”, “cell” or “cellular”, “systemic”, “biomedical” and “integrative” or “integrated”. Thirty-three departments were included in this subgroup. This included three departments that participated in multiple PhD programs, but had one program that met these criteria; only this program was used for subgroup analyses. Five departments offered general PhD programs in biomedical sciences, however the name of the concentration or track for physiology students met the criteria outlined above. Statistics requirements were similar in a sensitivity analysis that included only PhD programs in physiology (Statistics required: 63.6%, Recommended elective: 12.1%, Elective: 9.1%, Not required, not listed as an elective: 15.2%).

**References**

1. Weissgerber T, Milic N, Winham S, Garovic VD. Beyond Bar Graphs: Time for a New Data Presentation Paradigm. PLoS Biology. 2015;13: e1002128.
